# Utilization of a modified percutaneous nephrostomy tube placement: comparison to standard methodology

**DOI:** 10.1186/2036-7902-6-S1-A19

**Published:** 2014-01-31

**Authors:** L Proklova, TI Kyrkalova, DE Sablin, MU Yanitskaya

**Affiliations:** 1 Regional Children Hospital, Surgery Department, Arkhangelsk, Russian Federation

## Introduction

Percutaneous nephrostomy is used for temporary or permanent drainage of an obstructed pelvicalyceal system.

## Objective

The aim of this study is to compare the success rates and complications of the modified and standard technique in percutaneous nephrostomy tube placement (PCN) in dilated and non-dilated systems in children.

## Patients and methods

We retrospectively analysed 47 emergent percutaneous nephrostomies in dilated an non-dilated systems in 35 patients from 2001 to 2012 (range 1 months - 14 years), using standard or modified technique. “Standard” technique of PCN was performed using the Seldinger or one-step techniques in dilated and non-dilated systems under Ulrasound control. “Modified” technique was performed using Seldinger technique with insertion additional needle in pelvicalyceal system, and both the Ultrasound and Fluoroscopy control was used. Percutaneous nephrostomy was considered successful if catheter was placed in renal pelvis and drained urine spontaneously.

## Results

The success rates in cases with modified technique was 95.7%, and 80.7%, in those where standard technique was used. No complications were registered.

## Conclusion

Modified technique in percutaneous nephrostomy using additional needle and both the Ultrasound and Fluoroscopy control is successful and safe methodology in the temporary or permanent treatment of obstructed pelvicalyceal system in comparison with standard technique.

